# Immediate effects of binaural beats stimulation on prefrontal cortical activity in stroke patients: A pilot electroencephalogram study

**DOI:** 10.1097/MD.0000000000045784

**Published:** 2025-11-07

**Authors:** Ya-Ting Zhong, Shih-Fen Hsiao, Yu-Chen Lin, Chen-Wen Yen, Sing-Kai Lo, Jau-Hong Lin

**Affiliations:** a Department of Physical Therapy, College of Health Sciences, Kaohsiung Medical University, Kaohsiung, Taiwan; b Neurotechnology and Assistive technology Center, Kaohsiung Medical University, Kaohsiung, Taiwan; c Department of Mechanical and Electro-Mechanical Engineering, National Sun Yat Sen University, Kaohsiung, Taiwan; d Faculty of Liberal Arts and Social Sciences, Education University of Hong Kong, Hong Kong, China; e Department of Medical Research, Kaohsiung Medical University Chung Ho Memorial Hospital, Kaohsiung, Taiwan.

**Keywords:** binaural beat stimulation, music therapy, prefrontal cortex activity, stroke

## Abstract

Binaural beat stimulation (BBS) has been applied to explore cognitive and sleep disorders. This pilot study aimed to examine the immediate effects of a 4-Hz frequency BBS combined with music on prefrontal cortex activity in patients with stroke. We recruited 12 patients with chronic stroke from the rehabilitation department of a medical university-affiliated hospital. Each subject received “Musical Moonlight Sonata,” “4-Hz frequency BBS,” and “Musical Moonlight Sonata combined with 4-Hz BBS” interventions in a random order within a 3-hour experimental period. Each intervention lasted for 20 minutes, with 30-minute rest intervals between interventions. An 8-channel wearable dry electrode electroencephalography system was used to record the prefrontal cortex activity before and after each intervention. Within-group analysis revealed an increase in alpha power following: “Musical Moonlight Sonata” intervention (*R* = 0.57, *P* = .049). Significant differences in prefrontal cortical activity were found between the 3 interventions (partial *η*² = 0.24, *P* = .04). Compared to the “4-Hz BBS” intervention, the “Musical Moonlight Sonata” intervention significantly increased alpha power in the affected prefrontal cortex. Preliminary findings indicate that a 20-min intervention with Moonlight Sonata music alone had an immediate effect on prefrontal cortex activity in patients with chronic stroke. The 4-Hz BBS or the combined music and 4-Hz BBS intervention did not seem to be as effective in changing prefrontal cortex activity after stroke.

## 1. Introduction

Approximately 30% to 50% of stroke survivors experience emotional problems, such as depression and anxiety, limiting their social participation.^[1]^ Prolonged negative emotions increase cortisol levels and over-activate the sympathetic nervous system; this subsequently may elevate the heart rate and blood pressure, trigger chronic inflammation, and increase the risk of recurrent stroke.^[2–4]^ Understanding the emotional problems of stroke survivors is crucial for establishing effective treatment strategies.

Music therapy has been shown to improve negative emotions in stroke patients.^[5–7]^ Daily listening to favorite music for 30 minutes over a 2-month period was shown to achieve significant emotion improvement.^[5,7]^ Kim et al also reported mood improvements with music therapy.^[6]^ It was found that calming melodies such as Beethoven “Moonlight Sonata” could support emotion regulation.^[8]^ Hybrid music therapy has been used to enhance neurological and emotional recovery in stroke patients.^[9]^ Kiper et al demonstrated that music-integrated immersive virtual reality therapy significantly reduced depressive symptoms and improved daily living activities.^[10]^ Liu et al found that combining music therapy with robot-assisted rehabilitation could enhance upper limb motor function and improve emotional states.^[11]^ In another study, Liu et al showed that repetitive transcranial magnetic stimulation combined with music therapy improved language expression abilities and alleviated emotional distress in stroke patients.^[12]^ These findings suggest that the potential benefits of combining music therapy with new technologies may be higher than those of music therapy alone in stroke rehabilitation.

Binaural beat stimulation (BBS) is an auditory stimulus that may influence mood through brainwave entrainment.^[13]^ The brainwave entrainment hypothesis suggests that external auditory stimuli at specific frequencies can synchronize brain activity and induce distinct physiological and psychological states.^[14]^ Researchers have explored the BBS effects on various psychological phenomena linked to electroencephalogram (EEG) frequency bands, including affective states,^[15,16]^ mood,^[13,17]^ and relaxation.^[18]^ Previous studies have demonstrated that 4-Hz (theta frequency) BBS has a positive effect on emotional stability.^[14,17,19]^ Le Scouarnec et al found that 50 healthy adults who received 20 minutes of daily 4-Hz BBS for 4 weeks had a reduction in stress and anxiety levels.^[19]^ Wahbeh et al randomly assigned 60 university students to groups of 4-Hz BBS, 10-Hz BBS, and a relaxation training program, each receiving 30-minute daily sessions for 6 weeks. The 4-Hz BBS group attained the largest relaxation and anxiety reduction.^[17]^ In a study by Huang and Charyton, 30 insomnia patients who listened to 30 minutes of 4-Hz BBS before bedtime for 8 weeks reported earlier sleep onset and fewer nighttime awakenings.^[14]^ These studies suggest that a 4-Hz BBS provides a possible treatment strategy for emotion improvement.^[15]^ However, little is known about the effects of BBS in patients with stroke.

Although the 4-Hz BBS lies within the theta range, alpha activity (8–12 Hz) is more closely associated with affective regulation. Alpha power increases are widely regarded as neural markers of relaxation and peaceful states and processes that are closely related to emotional stability.^[20]^ Therefore, alpha power was selected as the primary EEG outcome to capture the emotion-related effects of the 4-Hz BBS in stroke patients. In the present study, we conducted an initial investigation of the immediate effects of 4-Hz BBS with or without music therapy on emotion regulation, as measured by EEG oscillatory activity in the prefrontal cortex after stroke. The research hypotheses were as follows:

(1) 4-Hz BBS alone could effectively promote prefrontal cortex activity on emotion regulation;
(2) Music therapy combined with 4-Hz BBS would produce an additional effect, leading to a greater impact than music alone or 4-Hz BBS alone.

## 2. Methods

The research protocol was approved by the ethics committee of the university hospital (KMUHIRB-F(II)-20220175). Informed consent was obtained from all the participants.

### 2.1. Participants

Patients with stroke were recruited from the rehabilitation department of a medical university-affiliated hospital. Inclusion criteria were as follows: age between 20 and 80 years; first-ever stroke confirmed by imaging study; unilateral hemiplegia over 6 months; Mini-Mental State Examination score ≥ 24;^[21]^ ability to sit independently for at least 30 minutes; and right-side dominance, indicating left hemisphere dominance. Patients with hearing impairment, aphasia with limited comprehension, pacemakers or other metallic implants, or any history of brain surgery were excluded. The research protocol was reviewed and approved by the Institutional Review Board of a medical university-affiliated hospital. All participants provided written informed consent before participating in the experiment.

### 2.2. Procedures

**Figure 1.**
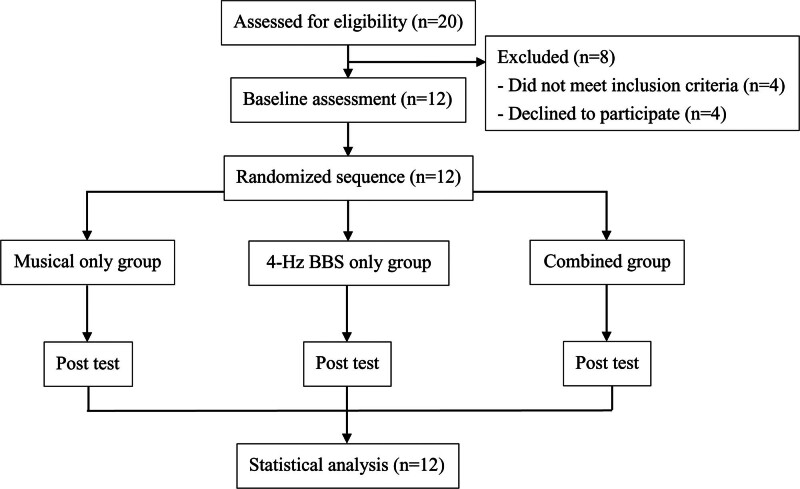
Study flowchart.

This study used a 1-group pre–post within-subject design (Fig. 1). Each subject received 3 interventions, namely, “Musical Moonlight Sonata,” “4-Hz frequency BBS,” and “Musical Moonlight Sonata combined with 4-Hz BBS,” in a random order within a 3-hour experimental period. Each intervention lasted for 20 minutes, with 30-minute rest intervals between interventions.

Participants sat in a quiet, controlled room (24–26°C, stable lighting). They avoided caffeine consumption and sleep deprivation before the experiment, and remained silent with their eyes closed during the testing. The participants sat on the same chair with a backrest for all intervention sessions and wore Bluetooth headphones with adjustable stimulation frequency modes (St. EEG, BIBE, Artise Biomedical Co., Ltd., Taiwan) to receive audio stimuli. The 4-Hz mode was chosen in this study for emotion improvement based on previous research protocols.^[15,17,19]^ The 3 intervention audio stimuli were: Beethoven Moonlight Sonata, First Movement only;^[8]^ 4-Hz BBS only; and Musical Moonlight Sonata combined with 4-Hz BBS.

An 8-channel wearable dry electrode EEG system (St. EEG Altaire, Artise Biomedical Co., Ltd, Taiwan) was used to assess prefrontal cortex activity before and after each intervention. A qualified physical therapist conducted the experimental process and evaluation.

### 2.3. Outcome measures

**Figure 2.**
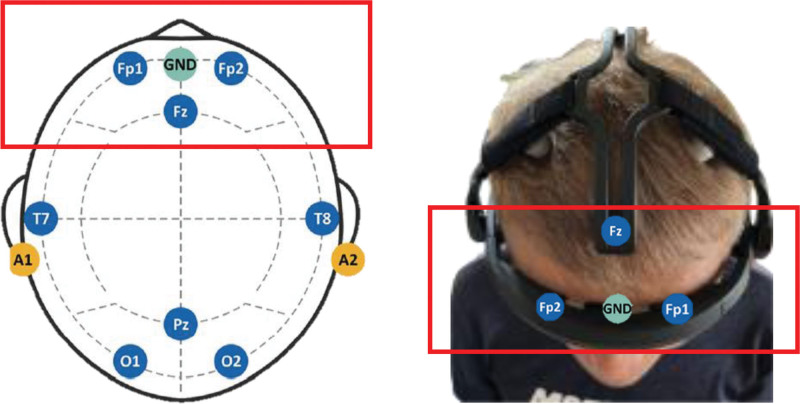
Eight-channel EEG system electrode positions. EEG = electroencephalogram.

Dry electrodes were placed according to the international 10 to 20 system, with 8 channels located at Fp1 (left prefrontal cortex), Fp2 (right prefrontal cortex), Fz (central frontal cortex), Pz (parietal cortex), O1 (left occipital cortex), O2 (right occipital cortex), T7 (left temporal cortex), and T8 (right temporal cortex).^[22]^ Additionally, 2 reference electrodes were placed on the left and right earlobes (A1 and A2) and a ground electrode, as shown in Figure 2.

After fitting the EEG equipment, electrode contact with the scalp was verified, and the impedance of each electrode was adjusted to below 300 kΩ. Raw EEG signals were recorded at a sampling rate of 1000 Hz. The signals were denoised and band-pass filtered using Python 3.8.10, to remove low-frequency baseline drift and high-frequency noise, and EEG signals in the 0.5 to 40 Hz range were retained. Spectral analysis was conducted using the Welch method to calculate the relative power of each frequency band, including alpha waves (8–13 Hz), low-beta waves (14–20 Hz), and high-beta waves (21–30 Hz).^[23]^ This normalization method was adopted to minimize the influence of inter-individual variability in absolute EEG amplitude, such as differences in scalp conductivity, electrode impedance, or baseline signal strength.^[24]^ An increased alpha wave power typically indicates enhanced relaxation and emotional stability.^[25]^

Primary measurements focused on alpha signals from Fp1 and Fp2 to assess changes in prefrontal cortex activity in the injured and non-injured brain regions. The change values represent the log-transformed ratio of the posttest to pretest mean power.^[25]^ A value greater than 0 indicates an increase in power.^[26]^ Alpha wave activities in these regions is closely linked to psychological stress and relaxation states.^[27,28]^ An increase in alpha power is typically interpreted as an indicator of a more relaxed mental state.^[29]^

**Figure 3.**
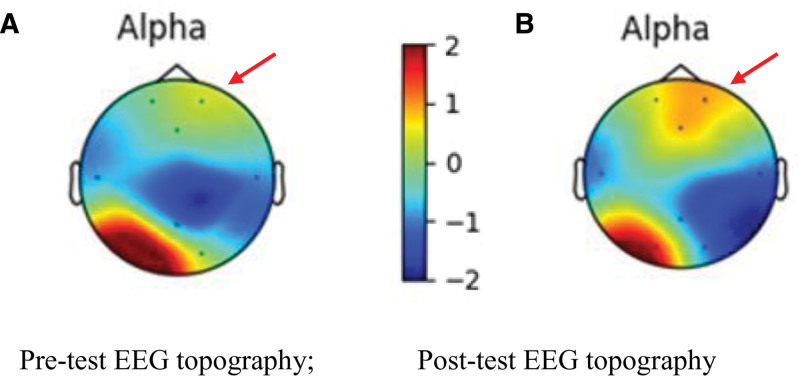
Sample EEG topography from participant 10 (A) before and (B) after the Musical Moonlight Sonata intervention. The color red indicates positive excitation with increased power values, while blue indicates negative inhibition with decreased power values. The arrow points to an increase in alpha wave values after the intervention (shown in orange-red), indicating activation of the prefrontal cortical area after the Musical Moonlight Sonata intervention in this subject. The color scale represents standardized *z*-scores, which have no units. The *z*-scores were calculated by the software using data from all EEG channels recorded for each participant. EEG = electroencephalogram.

EEG data were processed using the Cygnus Software (StEEG, Cygnus, St. EEG, Artise Biomedical Co., Ltd, Taiwan) to generate topographic maps displaying EEG changes, as shown in Figure 3.

### 2.4. Statistical analysis

Data were analyzed in SPSS 26.0 Statistical Software (IBM Corp., Armonk). In addition to descriptive statistics, the Shapiro–Wilk test was used to determine whether the EEG signal parameters were normally distributed. Pre- and post-intervention cortical activities for single interventions were not normally distributed, whereas changes in prefrontal cortical activity for the 3 interventions were normally distributed. Therefore, the Wilcoxon signed-rank test was used to compare pre- and post-intervention cortical activity for each intervention. One-way repeated-measures analysis of variance was used to compare changes in prefrontal cortical activity across the 3 interventions, with post hoc Bonferroni tests to identify the sources of differences. For effect size estimation, *r* was derived from the Wilcoxon signed-rank test (*r* = Z/√N), and partial *η*² was used for 1-way repeated-measures analysis of variance. Based on Cohen criteria, values of 0.10, 0.30, and 0.50 for *r*, and 0.01, 0.06, and 0.14 for partial *η*² represent small, medium, and large effects, respectively.^[30]^ Statistical significance was set at *α* < .05.

## 3. Results

This study recruited 20 stroke participants between April and June 2024. Eight participants were excluded because they did not meet the inclusion criteria or were unwilling to participate. No adverse effects or side effects were reported by the participants during the intervention and evaluation periods.

Table 1 presents the participants’ demographic and clinical characteristics. There were 8 males and 4 females, with an average age of 55.9 ± 11.2 years. Four participants had ischemic stroke and 8 had hemorrhagic stroke. Nine participants had left brain damage with right-sided hemiplegia, whereas 3 had right brain damage with left-sided hemiplegia. The average time since stroke onset was 43.9 ± 29.6 month.

**Table 1:** Demographic and clinical characteristics of the stroke participants.

No.	Age (yr)	Gender	Type of stroke	Injured brain region	Paretic side of limb	Months since stroke onset
1	61	Male	Hemorrhagic	Subcortical	Right	120
2	64	Male	Ischemic	Subcortical	Left	38
3	52	Female	Hemorrhagic	Subcortical	Right	32
4	57	Female	Ischemic	Brainstem	Right	31
5	46	Male	Hemorrhagic	Subcortical	Right	59
6	52	Male	Hemorrhagic	Brainstem	Right	70
7	44	Male	Hemorrhagic	Subcortical	Right	25
8	74	Male	Ischemic	Cortical	Left	35
9	58	Male	Hemorrhagic	Subcortical	Right	20
10	35	Female	Hemorrhagic	Cortical	Left	60
11	56	Female	Ischemic	Mixed	Right	13
12	72	Male	Hemorrhagic	Cortical	Right	24

Table 2 presents the pretest, posttest, and change scores of alpha power in the prefrontal cortex across the 3 interventions. The Musical Moonlight Sonata condition showed a trend toward increased alpha power in the injured hemisphere (*P* = .049). Between-group comparison revealed significant differences (partial *η*² = 0.24, *P* = .04), indicating a large effect size, with post hoc analysis showing that music intervention led to a greater alpha power increase than 4-Hz BBS.

**Table 2:** Pretest, posttest, and changes in alpha power of the prefrontal cortex with 3 interventions.

	Alpha (%)	Music alone	BBS alone	Combined	*F*	*P*
Injured brain	Pretest	0.0005 (0.0010)	0.0009 (0.0013)	0.0006 (0.0005)		
	Posttest	0.0013 (0.0015)*	0.0005 (0.0006)	0.0013 (0.0019)		
	Change	0.74 (0.92)†	-0.45 (1.31)†	0.14 (1.07)	3.45	*.04* * *
Non-injured brain	Pretest	0.0004 (0.0009)	0.0003 (0.0002)	0.0004 (0.0006)		
	Posttest	0.0010 (0.0015)	0.0004 (0.0006)	0.0009 (0.0024)		
	Change	0.02 (0.98)	-0.88 (1.72)	0.44 (1.45)	1.22	*.31*

Relative power (%) was used in the analysis, representing the proportion of alpha power to total electroencephalogram power; values are presented as mean (standard deviation).

The change value represents the log-transformed ratio of posttest to pretest mean power. A value >0 indicates an increase in power. Italicized *P* values represent the significance level of one-way repeated-measures ANOVA among the three interventions.

BBS = binaural beats stimulation, *F* = values are derived from a 1-way repeated-measures analysis of variance (ANOVA).

† A 20-minute music-alone intervention using the Moonlight Sonata had a greater immediate effect on prefrontal cortical activity than did BBS alone in individuals with chronic stroke).

* Wilcoxon signed-rank test results comparing pre- and post-intervention data within each condition (*P* < .05) indicated that a marginally significant within-group difference was found under the music alone condition.

** One-way repeated-measures ANOVA was used to compare differences among the 3 interventions, with Bonferroni post hoc tests to identify the sources of significant differences (*P *< .05) indicates significant differences among the 3 interventions.

## 4. Discussion

To our knowledge, no studies to date have examined the effects of the BBS on emotion regulation following stroke. The present study indicates that intervention with 20 minutes of music listening alone could potentially increase alpha power in the damaged prefrontal cortex. This suggests that the intervention had an immediate emotional relaxation effect and partially supported the notion that music intervention could promote relaxation and regulate emotions in patients with stroke. However, neither using 4-Hz BBS alone nor its combination with music produced a large enough additional effect compared to the music-alone intervention.

The prefrontal cortex is the core location of the brain, governing executive control, and cognitive function. This plays a critical role in the management of emotional responses. When individuals face stress or negative emotions, the prefrontal cortex helps regulate emotional reactions and maintain emotional stability.^[31]^ Studies have shown that individuals with emotional disorders typically exhibit lower prefrontal cortex activity and reduced ability to regulate emotional responses. These differences lead them to rely more on other brain regions under stress, and this shift further exacerbates emotional instability.^[32]^ In healthy individuals, the effects of music intervention are primarily achieved by reducing sympathetic nervous system activity and increasing parasympathetic nervous activity, thereby promoting relaxation.^[33,34]^ In stroke patients, music stimulation may enhance prefrontal cortex activity and improve the responsiveness of the nervous system.^[7]^ These differences suggest that the mechanisms of music intervention vary across populations.

Previous studies have shown that music interventions can increase alpha power by 18% to 25% in stroke patients.^[7,35]^ These findings suggest that classical music, meditation music, or other types of relaxing music can effectively enhance prefrontal cortex activity in patients with stroke, highlighting the potential benefits of music therapy in the recovery process after stroke. In this study, a single 20-minute intervention using the first movement of Beethoven Moonlight Sonata resulted in an increase in alpha power in the prefrontal cortex. These results suggest that music intervention has an immediate effect on reducing stress and stabilizing emotions during the recovery process in patients with stroke.

It is interesting to note that the responses to the Musical Moonlight Sonata intervention varied considerably among individuals in this study. While some participants demonstrated clear increases in alpha power within the injured hemisphere, others exhibited minimal or even slight decreases. This variability highlights the heterogeneity among patients with stroke, potentially reflecting differences in lesion location, neural plasticity, and responsiveness to auditory interventions.

Although this study did not find any noticeable effect of a 20-minute 4-Hz BBS intervention on prefrontal cortical activity in stroke patients, the results should be interpreted in the context of broader literature. A systematic review of 14 studies examined the effects of BBS ranging from 4 to 30 Hz on brain oscillatory activity, primarily in healthy adults and some patients with specific psychological conditions (e.g., depression and anxiety).^[36]^ The review revealed mixed findings: several studies reported clear entrainment of brain oscillations at the target frequency, supporting the hypothesis that BBS can influence neural activity,^[37,38]^ whereas other studies found no evidence of frequency-specific entrainment.^[39,40]^ Previous research has demonstrated that 4-Hz BBS has significant effects on promoting relaxation, reducing stress and anxiety, and improving sleep quality in healthy adults and patients with insomnia.^[14,17,19]^ These findings support the potential of the 4-Hz BBS as an effective, noninvasive method for promoting mental and physical health; it has been shown to enhance alpha power and improve psychological states.^[41,42]^ The mechanism involves influencing brainwave activity through auditory input systems, inducing oscillations at specific frequencies, and regulating emotional states.^[14]^ However, these studies primarily focused on healthy adults, with evaluations relying on functional scales and simple mental state examinations to assess cognitive changes under different binaural beat frequencies.^[37]^ These inconsistent findings may be attributed to several factors. First, there is substantial heterogeneity in study design, including differences in stimulation frequency, session duration, and EEG analysis methods. For instance, some studies have reported effects with longer exposure times or specific target frequencies,^[38]^ whereas shorter interventions or less optimal parameters showed weaker or null results.^[39]^ Second, the sample sizes were often small, limiting the statistical power and ability to detect subtle changes. Third, control conditions such as pink noise or other auditory stimuli sometimes produce similar changes in EEG activity, making it challenging to distinguish specific BBS effects from general auditory stimulation.^[40]^ Finally, participant characteristics likely play a role: most studies were conducted with healthy adults, whereas our study involved stroke patients with heterogeneous lesion locations and variable neural responsiveness, which could make entrainment more difficult to observe.

As this study was the first to investigate the immediate effects of 4-Hz BBS either in combination with or without music in stroke patients, we showed that although the study hypothesis was not fully supported, the setup of the experiment and assessment protocol of prefrontal cortical activity using simplified EEG was feasible, with good tolerance and no side effects. Researchers may consider adopting the same or similar intervention duration and frequency of BBS in future studies.

Previous studies have found that combining music therapy with other interventions generally yields better results than music therapy alone.^[10–12]^ In this study, we did not find such an effect. Apart from the difference in the intervention parameters, the discrepancy may also have been caused by the wide variation in the brain regions affected. Music processing involves multiple brain areas such as the frontal lobe, temporal lobe, and limbic system. In stroke patients, lesions in these areas may influence the neural response to musical stimuli and subsequently affect the effectiveness of therapy.^[40,43]^ Our study suggests that individuals with subcortical or cortical lesions, as well as those with right hemisphere lesions, exhibit greater sensitivity to alpha power modulation, particularly in response to music alone or BBS-alone interventions. In contrast, individuals with brainstem or mixed lesions and those with left hemisphere lesions showed limited responsiveness, with minimal or no substantial modulation. These observations imply that the structural integrity of the subcortical and cortical regions, along with the functional specialization of the right hemisphere, may play a critical role in supporting neurophysiological responses to auditory or combined auditory stimulation therapies. Future research could further explore the impact of brain lesion location on the effectiveness of music therapy to optimize intervention strategies.

As a feasibility project, the present study has a few limitations. First, the small sample size and high heterogeneity of participants affected the interpretative power of the statistical tests and generalizability of the results. Future studies with more participants are necessary to confirm the effects of BBS in suitable patients with stroke. Second, we only observed immediate short-term effects with the 20-minute single-session intervention. It is not known whether using a longer duration for a single session or an intervention consisting of repeated sessions will make any difference in immediate or long-term outcomes. Third, the bluetooth headphones used in this study had only 3 BBS frequencies (2-Hz, 4-Hz, and 8-Hz), which limited the utilization of a wider delivery frequency range and may also have limited therapeutic effects. Future research should integrate smartphone apps for the real-time control of BBS and frequency ranges to investigate the effects of different frequencies. Fourth, no silent condition or healthy control group was included, which limits direct comparisons and generalizability of the findings. Future studies with extended designs should incorporate control conditions and healthy participants to provide a more comprehensive understanding of the effects of BBS after a stroke. Finally, no subjective data regarding participants’ emotional experiences were collected. As this was a pilot study, we prioritized objective EEG measurements to reduce the participant burden and session complexity. Future studies should incorporate standardized self-report questionnaires or interviews to better understand the relationship between cortical changes and emotional outcomes.

## 5. Conclusion

This pilot study found that a 20-minute music-alone intervention using Moonlight Sonata had an immediate effect on prefrontal cortical activity in chronic stroke patients. Combining the same music with 4-Hz BBS or using 4-Hz BBS alone as audio stimuli did not show any immediate change in prefrontal cortical activity in stroke patients after the same duration of intervention.

## Acknowledgments

The authors thank the participants for their participation in this study.

## Author contributions

**Conceptualization:** Shih-Fen Hsiao, Chen-Wen Yen, Jau-Hong Lin.

**Data curation:** Ya-Ting Zhong, Jau-Hong Lin.

**Formal analysis:** Yu-Chen Lin, Jau-Hong Lin

**Funding acquisition:** Jau-Hong Lin

**Investigation:** Ya-Ting Zhong.

**Methodology:** Shih-Fen Hsiao, Yu-Chen Lin, Chen-Wen Yen, Sing-Kai Lo, Jau-Hong Lin

**Project administration:** Jau-Hong Lin

**Resources:** Jau-Hong Lin

**Software:** Chen-Wen Yen

**Supervision:** Jau-Hong Lin

**Writing – original draft:** Ya-Ting Zhong.

**Writing – review & editing:** Shih-Fen Hsiao, Sing-Kai Lo, Jau-Hong Lin
